# Role of Circulating microRNAs in Liver Disease and HCC: Focus on miR-122

**DOI:** 10.3390/genes15101313

**Published:** 2024-10-12

**Authors:** Francesco Colaianni, Veronica Zelli, Chiara Compagnoni, Martina Sara Miscione, Mario Rossi, Davide Vecchiotti, Monica Di Padova, Edoardo Alesse, Francesca Zazzeroni, Alessandra Tessitore

**Affiliations:** Department of Biotechnological and Applied Clinical Sciences, University of L’Aquila, Via Vetoio, 67100 L’Aquila, Italy; francesco.colaianni1@graduate.univaq.it (F.C.); chiara.compagnoni@univaq.it (C.C.); martinasara.miscione@graduate.univaq.it (M.S.M.); mario.rossi@univaq.it (M.R.); davide.vecchiotti@univaq.it (D.V.); monica.dipadova@univaq.it (M.D.P.); edoardo.alesse@univaq.it (E.A.); francesca.zazzeroni@univaq.it (F.Z.); alessandra.tessitore@univaq.it (A.T.)

**Keywords:** miRNAs, miR-122, diagnostic biomarkers, liver disease, NAFLD, HBV, HCV, HCC

## Abstract

miR-122 is the most abundant microRNA (miRNA) in the liver; it regulates several genes mainly involved in cell metabolism and inflammation. Host factors, diet, metabolic disorders and viral infection promote the development of liver diseases, including hepatocellular carcinoma (HCC). The downregulation of miR-122 in tissue is a common feature of the progression of liver injury. In addition, the release of miR-122 in the bloodstream seems to be very promising for the early diagnosis of both viral and non-viral liver disease. Although controversial data are available on the role of circulating miR-122 as a single biomarker, high diagnostic accuracy has been observed using miR-122 in combination with other circulating miRNAs and/or proteins. This review is focused on comprehensively summarizing the most recent literature on the potential role of circulating miR-122, and related molecules, as biomarker(s) of metabolic liver diseases, hepatitis and HCC.

## 1. Introduction

MicroRNAs (miRNAs) are small non-coding RNAs able to regulate gene expression at the post-transcriptional level, involved in many fundamental physiological and pathological processes, including cell survival and proliferation, metabolism, inflammation and carcinogenesis. MiRNAs can be found in tissues as well as in body fluids such as plasma/serum, where they are extraordinarily stable due to their nature and structure [1]. In fact, circulating miRNAs exist in association with RNA-binding protein or encapsulated in nanoparticles, as exosomes, apoptotic bodies and micro-vesicles, with consequent preservation increase [2]. In the last years, circulating miRNAs have been extensively studied as putative biomarkers for the diagnosis of different cancers, including hepatocellular carcinoma (HCC). In addition, their release into the bloodstream can be considered also related to different types of liver disease [3,4]. 

MiR-122 is a liver-specific miRNA, whose physiological and pathological function was extensively described and characterized in liver tissue. Dysregulation of miR-122 expression levels was observed in almost all cancer types, as it seems to be a crucial component in tumor growth, and recent data show that miR-122 plays a role as an oncogenic or tumor suppressor miRNA, depending on the tumor type [5]. MiR-122 is the most abundant miRNA in the liver, mainly involved in liver metabolism and inflammation [6]. It also plays a role in different pathogenetic mechanisms, as hepatitis C (HCV) and hepatitis B (HBV) virus infection [7], metabolic liver disease, including non-alcoholic fatty liver disease (NAFLD), alcoholic liver disease (ALD) and cancer [8]. 

Here, we summarize the current knowledge on the function of miR-122 as a regulator of liver metabolism and its dysregulation in pathological conditions, with a specific focus on the potential role as a circulating biomarker of liver disease onset and progression up to HCC development. Notably, the current manuscript is based on the most recent literature (2019–2024) regarding miR-122-5p (aliases miR-122, miR-122a), which is the most studied form of mir-122.

## 2. Epidemiology and Risk Factors of Liver Disease and Hepatocellular Carcinoma

HCC is the fourth most common cause of death in 90 countries worldwide, accounting, in 2020, for over 800,000 deaths [9]. In addition, HCC incidence and related mortality is expected to increase by more than 50% over the next 20 years [10]. Africa and Asia represent 80% of all HCC cases, while in Europe and America, the disease is less frequent [11].

The global burden of chronic liver disease is still increasing and is strongly linked to HCV and HBV viral infections, especially in Asian and African populations, as they are the most affected by liver cirrhosis [12]. On the other hand, HBV and HCV prevention is reflecting a decrease in the burden of liver cancer in Europe and America, while the prevalence of other risk factors is increasing [10]. Hence, the landscape of HCC etiology is changing from viral infection to diet-related risk factors and metabolic disorders.

Overall, HBV and HCV remain the most important risk factors for HCC development in Asian countries, where about 80% of cases are mainly related. The European population has the highest alcohol consumption rate, known to be an important risk factor for the development of ALD. In Latin America, 48% of cases are due to HCV infection, followed by 22% related to ALD [13]. The incidence of ALD is also increasing in the USA, where liver disease is currently mainly related to obesity, diabetes and a fat-rich diet (e.g., western style), which promote liver steatosis and glucose/fructose metabolism disorders in the hepatocytes [14]. In particular, metabolic disease reflects damage characterized by a large spectrum of metabolic disorders: among them, type 2 diabetes, dyslipidemia, obesity and hypertension are the main contributing factors to the etiology of metabolic liver diseases. All of them contribute to the development of NAFLD, which includes steatosis (NAFL), non-alcoholic steatohepatitis (NASH) and liver cirrhosis [15], and is characterized by initial lipid accumulation in hepatocytes, followed by chronic inflammation, fibrosis and severe damage leading to HCC in a percentage of cases [16].

Host factors and genetic susceptibility are other important risk factors associated with HCC development. Host susceptibility factors are older age (median age at HCC diagnosis is 60 years, with the highest incidence between 60 and 74 years) and sex. Men are more susceptible to HCC development than women. In fact, some studies reported that the estrogen axis modulates the inflammatory microenvironment, thus protecting against HCC [13].

## 3. Current Diagnostic Methods of Liver Disease

Liver function tests, which rely on higher aminotransferases levels in blood, provide information about diet alteration, such as alcoholic abuse and a fat-rich diet. Generally, the aspartate transaminase (AST)/alanine transaminase (ALT) ratio and carbohydrate-deficient transferrin (CDT) can be used to detect ALD and chronic alcohol consumption. However, they provide very little information regarding fatty-liver alteration and early-stage NAFLD for the definitive diagnosis of which current guidelines recommend histological analysis following liver biopsy, which can cause, unfortunately, possible relevant complications [17,18]. In this context, the European Association for the Study of the Liver (EASL) also suggests the use of magnet resonance imaging (MRI) using proton density fat fraction (PDFF), which is considered as similar to a biopsy in terms of results but with a high cost and time consuming [19]. Another frequently used imaging approach is the inexpensive, available, free of possible side effects, repeatable sonography, even considering its technical developments in recent years [20]. In addition, guidelines recommend non-invasive blood-based tests to screen patients with liver disease (e.g., NAFLD), such as Fibrosis-4-Index (FIB-4) and NAFLD Fibrosis Score (NFS). However, these methods show a high risk of false positive results for more advanced fibrosis and reduced performance for obese or diabetic patients [19]. Another commonly used serum biomarker is Alpha Fetoprotein (AFP) that is not precisely diagnostic but mainly suggestive of HCC occurrence [21,22]. Nevertheless, to date, liver biopsy is still considered the best diagnostic method.

Several studies demonstrated that the combination of several serum biomarkers in panels can increase their diagnostic performance. Therefore, in the future, a combined approach could be used in clinical applications [23].

HCC diagnosis and management could be complicated by liver viral infection because HBV and HCV infections are related to the development of HCC. A viral chronic hepatitis diagnosis is based on viral load, antigens’ analysis for HBV and anti-HCV antibodies for HCV. However, there are no specific biomarkers that provide information on the risk of developing HCC in patients with viral liver infection. In addition, serum AFP has a limited sensitivity and specificity because the different etiology of liver disease could reduce AFP release from the liver [21,24]. The main diagnostic methods currently available are reported in Table 1.

In this context, there is an urgent need to identify novel biomarkers which, alone or in combination with others, can give information for the earlier diagnosis of liver disease. Circulating miRNAs are emerging as promising non-invasive diagnostic biomarkers in different types of disorders. To date, several miRNAs have been suggested as potential biomarkers in different types of liver diseases [25,26].

Among the candidate circulating miRNAs, the literature indicates that miR-122 may be considered one of the most promising [3,27].

## 4. MiR-122: Biogenesis and Role in Liver Physiology and Disease

MiRNAs are small non-coding RNAs, 21–30 nucleotides in length, that are able to regulate gene expression at the post-transcriptional level. In humans, more than 15,000 genes are modulated by more than 2600 miRNAs [28]. Notably, a single miRNA can modulate tens of mRNAs, and one single mRNA can be regulated by multiple miRNAs [29,30]. MiRNAs negatively regulate target genes by binding to the mRNA 3′ untranslated region (UTR). In cancer, depending on the function of related target genes, miRNAs can be considered as oncomiR or tumor suppressor miRNAs, thus promoting or suppressing oncogenesis processes [31,32].

The MiR-122 gene is located on chromosome 18, both in *Homo sapiens* and *Mus musculus*, and it is transcribed by RNA polymerase II. The pri-miR-122, 4.5 kb in length, is processed to the 66 nucleotides in length pre-miR-122. Mature miR-122 is 22 nucleotides in length (https://www.ncbi.nlm.nih.gov/gene/?term=hsa-miR-122, accessed on 15 May 2024).

MiR-122 is the most abundant miRNA in the liver and is involved in the regulation of multiple biological processes [6,33]. In particular, miR-122 suppresses several non-hepatic genes in the liver and its expression in human stem cells promotes differentiation to hepatocytes, confirming its function as a modulator of the differentiation at the level of the liver [34,35]. Furthermore, liver metabolism is linked to miR-122 expression and regulation, as miR-122 modulates several target genes, including lipogenic and glucogenic enzymes, channels and transporters, via different pathways. In this context, the most characterized target genes of miR-122 belong to the solute carrier family (*SLC*), among them *SLC7A1* [6]. In addition, miR-122 regulates metabolic processes via targeting the Aldolase A, Fructose-Bisphosphate (*ALDOA*) enzyme to prevent hypoxia-associated metabolism, which is a common feature of the progression of HCC [36]. According to Long et al., miR-122 modulates the lipogenesis by targeting Sirtuin-1 (*SIRT1*), leading to the upregulation of lipogenic enzymes and increase in lipids droplets in hepatocytes [8]. Silencing of miR-122 leads to decreased serum cholesterol levels in mouse models, suggesting that miR-122 target genes are involved in cholesterol biosynthesis and transport [35]. MiR-122 is deeply involved in liver homeostasis through the modulation of liver inflammation, as miR-122-KO mice show inflammatory phenotypes and increased hepatocarcinogenesis [33]. MiR-122 modulates cell migration by targeting LamininB2 (*LMNB2*) and the Disintegrin and Metalloprotease family 10 and 17 (*ADAM10, ADAM17*), and inhibits epithelial–mesenchymal transition, a common feature of HCC [37,38]. In addition, miR-122 controls cell proliferation by targeting factors such as the serum response factor (*SRF*), Insulin-like Growth Factor Receptor 1 (*IGFR1*), Cell Division Cycle 6 protein (*CDC6*), Minichromosome Maintenance Complex Component 4 (*MCM4*) and Cyclin G1 (*CCNG1*) [33,38,39,40].

Furthermore, miR-122 represents an essential host factor for HCV infection; it induces HCV-RNA translation because it can bind the HCV genome at different levels by unfolding the internal ribosome entry site. In addition, it protects the 5′-untranslated region (UTR) of the HCV genome by exonuclease activity and stabilizes it during the viral infection [7,41]. MiR-122 also appears to be partially involved in HBV infection, in this case by restricting viral replication. Indeed, the downregulation of miR-122 in tissue, induced by the HBV-X protein, promotes HBV infection and replication [42].

**Figure 1:**
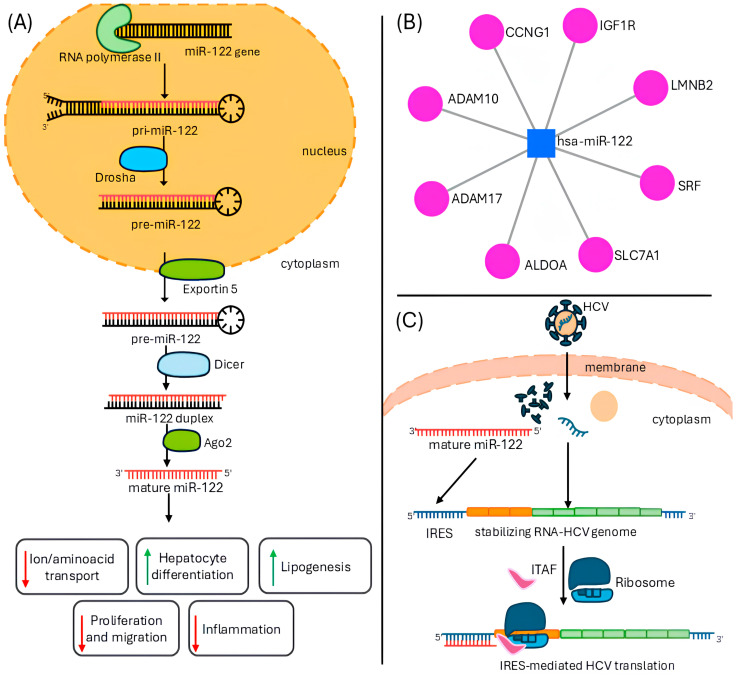
Representation of miR-122 biogenesis and function in the liver. (**A**) Mature miR-122 modulates hepatocyte differentiation, inflammation. Amino acid and ion transport, cell proliferation and migration, and promotes lipogenesis. (**B**) Picture obtained from miRNET database (https://www.mirnet.ca/, accessed on 8 September 2024) showing the main experimentally validated target genes of miR-122. In particular, miR-122 regulates cell proliferation and migration by interacting with *LMNB2*, *ADAM10*, *ADAM17*, *CCNG1*, *SRF* and *IGF1R* mRNA. MiR-122 regulates metabolism and ion/amino acid transport by interacting with *ALDOA* and *SLC7A1* mRNA. (**C**) During HCV infection in hepatocytes, mature miR-122 stabilizes HCV genome by interacting with IRES at 5′-end and promotes viral genome translation.

The main molecular and functional characteristics of miR-122 described above are summarized in Table 2 and Figure 1.

Since miR-122 is strongly involved in the regulation of morphological and functional features in a healthy liver, its dysregulation can be related to the development of liver disease. In particular, miR-122 downregulation is a common feature of different types of liver disease and HCC, as the lack of miR-122 induces abnormal fatty acid accumulation and promotes cell proliferation and migration, thus ascribing it the role of tumor suppressor miRNA in the liver [35,37,43].

Furthermore, several recent studies have reported that miR-122 is released into the bloodstream during the development of liver diseases, including HCC [27,44]. Elevated circulating levels of miR-122 seem to correlate with early liver disease, as metabolic syndrome and NASH, whereas, during the progression to cirrhosis, circulating miR-122 seems to decrease [26]. In addition, miR-122 was found to be upregulated in sera from viral hepatitis patients [45,46]. Hence, a circulating miR-122 level increase could indicate early liver disfunction and viral infection.

Notably, circulating miR-122 is probably liver-derived, due to its enriched expression in hepatocytes, and released upon liver injury [47]. Furthermore, although circulating miRNAs certainly represent a cross-talk system between cells and tissues, the importance and specific function of circulating miR-122 is not fully understood [26].

To date, most papers have mainly focused on the role of circulating miRNA-122 levels as potential diagnostic, prognostic and/or predictive biomarkers rather than on its biological functions.

In the following sections, we will discuss the most recent literature regarding the potential role of miR-122 as a circulating biomarker of liver disease, with a particular focus on metabolic liver diseases, hepatitis and HCC.

## 5. MiR-122 as Biomarker of Metabolic Liver Disease

NAFLD is considered among the most common chronic liver diseases worldwide. It shows a 25% prevalence in the adult population and can induce serious liver damage through the progression, in approximately 10% of cases, from simple steatosis to NASH, cirrhosis and HCC [48]. In this context, there is a strong need to identify high-risk patients for these possible severe complications. Although several non-invasive tests have been developed (e.g., FIB4 and NFS), it would be very useful to identify new biomarkers, not only to ascertain NAFLD diagnosis but also to understand its likely progression. Regarding the most recent literature, several studies strongly support the possible use of miR-122, alone or in association with other miRNAs, to reinforce NAFLD diagnosis and prognosis.

Cai et al. provided a systematic review and meta-analysis considering the relevant literature from 2000 to 2019: 17 studies comprising more than 1400 patients and 900 healthy people were the subject of analysis. The authors concluded that, within a panel of several circulating miRNAs, miR-122 upregulation showed high NAFLD diagnostic accuracy [49].

Similarly, Oses et al. systematically reviewed circulating miRNA profiling studies on children and adolescents with obesity and/or associated metabolic disorders and described miR-222, miR-142-3, miR-140-5p and miR-143 as overexpressed in obese children, whereas miR-122 and miR-34a were overexpressed in children with NAFLD and/or insulin resistance, suggesting possible miRNA associations to discriminate adolescents based on obesity-related diseases [50].

Recently, Li et al. performed a study on 81 and 54 adolescents with and without NAFLD, respectively, all undergoing bariatric surgery due to severe obesity. A total of 843 plasma samples were collected and miRNAs were profiled: five miRNAs (miR-122-5p, miR-1343-5p, miR-193a-5p, miR-193b-5p and miR-7845-5p) were found to be positively associated with histological NAFLD features [51].

In a group of 62 female patients stratified based on different combinations of obesity, body mass index (BMI), blood glucose and fatty liver levels, Pillai et al. showed significant expression increases in miR-122, miR-34a, miR-16, miR-21 and miR-375 in diabetic and obese as well as diabetic with steatosis patients with respect to controls. The same trend was observed for plasma IL-6, Leptin and FABP-1, whereas adiponectin showed an opposite behavior [52].

Furthermore, in a study on sera from an NAFLD high-fat diet (HFD)-fed mouse model, NAFLD patients and healthy individuals, Jiang et al. detected an miR-122 expression increase in serum and circulating extracellular vesicles (EVs) obtained from HFD mice and NAFLD patients [53].

Several recent studies also evaluated the potential role of miR-122 as a prognostic biomarker in relation to NAFL progression and patient survival.

Samy et al. reported exosomal miR-122, miR-128, miR-200, miR-298 and miR-342 expression changes both in NAFL and NASH patients in comparison to normal subjects. Such miRNAs were also in association with changes in other factors, such as FoxO3, adiponectin, PPAR-γ, LPS and TLR-4. MiR-122, miR-200, miR-298 and miR-342 showed the best receiver operating characteristic (ROC) curves in terms of NASH diagnostic markers [44].

In a study by Ezaz et al. on 132 subjects with diagnosed NAFLD, a strong association between miR-34a, miR-192, miR-200a and miR-122 serum levels and NAFLD severity was proven. Expression of miR-122, miR-34a and miR-192 was related to pathogenic factors (e.g., insulin resistance, two polymorphisms at the level of a patatin-like phospholipase domain containing 3 and transmembrane 6 superfamily 2) as well as steatosis and fibrosis, but not to inflammation or ballooning. On the other hand, the miR-200a level was associated only to fibrosis and to the transmembrane 6 superfamily 2 polymorphism. This study indicates that the four miRNAs could be used as putative biomarkers for NAFLD progression [54].

Lopez-Pastor et al. used an apolipoprotein E-deficient HFD-fed mouse model to understand the role of circulating miRNAs in NAFLD progression and identified level increases in miR-27b-3p and miR-122 from EVs in both Apoe^−/−^ HFD and standard diet (SD)-fed mice compared to (SD)-fed WT mice [55].

Newman et al. quantified the expression levels of miR-122, miR-192 and miR-128-3p in liver-specific EVs isolated by anti-Asialoglycoprotein Receptor 1 (ASGR1) immunoprecipitation, global EVs and total cell-free RNA obtained from NAFL, NASH and healthy controls. Increased levels of all three miRNAs during disease progression were observed only in ASGR1 EVs from NASH patients with respect to healthy controls, but not in total cell-free RNA or global EVs [56].

Notably, a longitudinal analysis of serial liver biopsies from Japanese NAFLD patients highlighted circulating miR-122 as a dynamic prognostic factor for individuals showing severe liver fibrosis, and lacking stage scores’ improvement. In fact, at first liver biopsy, patients who developed cancer and those who did not both showed similar serum miR-122 levels. Conversely, at the second liver biopsy, patients who developed liver cancer showed lower miR-122 levels [57].

Finally, regarding the prognostic value of miR-122 in predicting patient survival, Akuta et al. described miR-122 and FIB-4 index serum levels as predictive risk factors of mortality. They analyzed 392 sera from Japanese patients with an NAFLD diagnosis and defined three different subgroups, depending on miR-122-fold change (FC) in serum levels (FC < 0.5, 0.5 < FC < 1, FC > 1). Results highlighted that the higher the miR-122 FC, the better the cumulative survival rate, suggesting that a circulating miR-122 levels’ decrease could be associated to liver disease aggressiveness [58].

Circulating miR-122 expression is also dysregulated in ALD, a disorder starting with simple fatty liver and progressing to alcoholic hepatitis, fibrosis and cirrhosis up to liver metabolic functioning failure and HCC. A very recent meta-analysis examined sera from alcohol-consuming individuals and ALD patients and found that miR-122 and miR-155 were upregulated in comparison with people with no alcohol use and healthy controls. On the other hand, only miR-122 levels were increased in patients with alcoholic hepatitis, indicating that miR-122 could be considered one of the most promising biomarkers for ALD diagnosis [59].

To summarize, the upregulation of miR-122 circulating expression levels in NAFLD and ALD is widely described, and increasing evidence supports its potential role, alone or in combination with other miRNAs/proteins, as an early diagnostic marker of metabolic liver disease. On the other hand, further studies are needed to make clear the potential role of miR-122 as a prognostic biomarker of disease progression and survival.

## 6. MiR-122 as Biomarker of Hepatic Viral Infection

To date, HCV and HBV infection represents a serious concern for public health, showing a high incidence and prevalence, especially in Asian and African populations, as no prevention methods are currently available [12].

To assess the diagnostic potential of circulating miR-122 levels in detecting patients with HBV- and/or HCV-associated chronic viral hepatitis, Zhou et al. performed a meta-analysis, including a total of 15 studies. Results showed an overall very good diagnostic accuracy, slightly higher in HCV- than HBV-related patients [60].

Elfert et al. reported upregulation of miR-122 in sera from 90 HCV Egyptian patients compared to 60 healthy donors, highlighting its ability to discriminate with excellent accuracy the two groups [45].

Overall, the literature shows consistent results on increased expression levels of circulating miR-122 in HCV-related patients compared to healthy controls [61,62,63] as well as its promising diagnostic potential, which seems higher than routine liver function tests, such as ALT/AST enzyme measurement [61].

Notably, Ullah et al. performed a study to profile miR-122 expression by stratifying patients according to the severity of HCV infection. MiR-122 circulating levels were upregulated in HCV patients (mainly genotype 3) with mild, moderate and severe cirrhosis compared to controls. In this study, patients with mild cirrhosis showed the most markedly increased miR-122 expression levels. ROC curve analysis also revealed a good diagnostic potential for miR-122 in discriminating between healthy controls and all three groups of chronic HCV patients (mean AUC 0.82, *p* < 0.0001) [47].

Similarly, El-Ahwany et al. analyzed miR-122 expression levels in sera of HCV genotype 4 Egyptian patients at different stages of liver disease. Comparing HCV fibrosis patients and HCV cirrhosis patients, miR-122 was found to be upregulated in both subgroups of HCV-infected patients with respect to healthy controls [64].

Taken together, data suggest that miR-122 expression levels are significantly increased in HCV patients compared to healthy controls and could represent a promising diagnostic biomarker, regardless of liver injury stage and HCV genotype.

In addition to its diagnostic potential, some authors also described circulating miR-122 as a potential predictor of therapeutic response and useful biomarker of liver damage progression after therapy in HCV patients and HCV/HIV co-infected patients.

Analyzing pretreatment serum samples from 160 chronic HCV genotype-4 patients and 50 healthy controls, Hassuna et al. evaluated the potential role of four liver-associated miRNAs (miRNA-122, miR-155, miR-196 and miR-29) as circulating biomarkers for the diagnosis and prediction of the response to direct-acting antiviral (DAA) treatment. MiR-122 was found to be upregulated in HCV patients compared to controls, showing a good diagnostic accuracy and a positive strong correlation with viral load (r = 0.856, *p* < 0.001). Furthermore, pretreatment miR-122 expression levels were significantly lower in DAA therapy-responder compared to non-responder patients, emerging as a valuable predictive biomarker of therapy response [65].

Recently, Ezzat et al. found a significant decrease in serum levels of miR-122, miR-221, miR-23a, miR-125, miR-217 and miR-224 in HCV patients after 12 weeks of DAA treatment, linking the dysregulation of these miRNAs to the dynamics of chronic HCV infection and thus suggesting their potential role as predictive biomarkers for HCV clearance [66].

Through the analysis of patients with HIV-1/HCV co-infection at baseline of DAA therapy and 12 weeks after treatment, Franco et al. observed that circulating levels of miR-122, as well as miR-100-5p_iso3p:-2 and miR-192-5p, were significantly decreased in therapy-responder patients compared to non-responders [67].

Furthermore, Muthukumar et al. conducted a pilot study enrolling allograft-treated patients characterized by non-viral liver disease or HCV. Their findings suggested that serum miRNAs’ quantification could be important for the diagnosis of recurrent hepatitis C virus (R-HCV) and non-viral acute rejection (NINV-AR). This model identified miR-122, miR-34a and miR-210 as putative predictive biomarkers to detect and discriminate AR from R-HCV [68].

Regarding the role of circulating miR-122 in HBV infection, less data are currently available.

Cavallone et al. performed circulating miRNA sequencing in chronic hepatitis B and HBeAg-negative-HBV infection and, among the differentially expressed miRNAs, found that miR-122-5p was significantly upregulated in HBV patients compared to controls [69].

Mokhtari et al. analyzed the expression levels of miR-122, miR-583 and miR-24 in chronic HBV patients, considering both active HBV (ACH) patients and inactive carriers (IC), and healthy controls. They found a significant increase in the circulating levels of miR-122 and miR-583 in the whole series of HBV patients compared to controls as well as a significant increase in expression levels of miR-583, but not miR-122, in ICs compared to ACHs. MiR-122 also emerged as a valuable diagnostic biomarker, able to discriminate with good accuracy HBV patients [AUC 0.89, *p* < 0.0001] [70]

Liu et al. found an upregulation of serum miR-122 expression levels mainly in HBV-infected patients and, to a lesser extent, in chronic HBV carriers, compared to healthy controls as well as in HBeAg-positive vs. HBeAg-negative patients [71].

Overall, similar to what was observed in HCV-infected individuals, miR-122 showed increased expression levels in HBV patients compared to healthy controls, regardless of the stage of liver injury.

Notably, the most recent studies mainly focused on miR-122 as a putative biomarker of liver disease progression, in the context of HBV-associated HCC. The discussion of data is provided in the following section.

## 7. MiR-122 as Biomarker of HCC

MiR-122 is dramatically downregulated in liver cancer tissue, resulting in the promotion of inflammation and cell cycle progression [41].

Conversely, circulating miR-122 levels appear to be generally increased in HCC patients compared to healthy controls and showed good performance parameters for HCC diagnosis. However, data on miR-122 expression levels in HCC compared to other liver diseases, in particular viral hepatitis as well as considering the different HCC etiology, are more controversial.

Zhao et al. provided a meta-analysis that summarizes the results from 13 studies yielding data for 920 HCC patients and 1217 controls. According to the authors, miR-122 could be useful for distinguishing HCC from healthy controls (AUC 0.91, *p* < 0.01) and from HBV or HCV infection (AUC 0.87, *p* < 0.01), but less efficient in discriminating HCC patients from those with liver cirrhosis or dysplastic nodule formation (AUC 0.74, *p* < 0.01). The authors suggested that serum miR-122 may be of great value for HCC surveillance, even if additional strategies are required for the detection of liver cirrhosis or liver dysplastic nodules. However, the heterogeneity of studies analyzed, for example, in terms of differences in patient characteristics and etiology of HCC, which were not considered here, is one of the main limitations of this meta-analysis [72].

Fang et al. reported significant higher expression levels of miR-122 and miR-16 in 100 early-stage HCC patients compared to 100 controls, which included HBV patients, liver cirrhosis, liver metastases, hepatic hemangioma and healthy individuals. Both miRNAs showed valuable diagnostic parameters in discriminating HCC patients from healthy controls and patients with other liver diseases (AUC 0.759 *p* < 0.001 and AUC 0.798 *p* < 0.001 for miR-122 and miR-16, respectively), further improved by combining the two miRNAs with AFP (AUC 0.862, *p* < 0.001). Notably, when compared with each subgroup of the control series, miR-122 was significantly higher in HCC patients compared to liver metastases, HBV and healthy controls, but not compared with liver cirrhosis and hepatic hemangioma. Furthermore, miR-122 was significantly higher in HBV patients than healthy controls, suggesting a gradual increase in miR-122 expression levels from healthy subjects to HBV patients up to HCC [73].

Pelizzaro et al. carried out a case study to determine the efficacy of miR-122 and miR-21 as diagnostic and prognostic biomarkers in HCC patients under treatment with Transarterial Chemoembolization (TACE). Through the analysis of healthy subjects, cirrhotic patients and HCC patients (tested before and after TACE), they observed a progressive miR-122 increase from controls to cirrhosis up to HCC (before TACE) as well as a further increase after treatment. MiR-122 levels were also associated with HCC etiology, being significantly higher in HCV/HBV-related patients compared to patients with other etiologies, including ALD [74].

Similarly, Mjelle et al. found miR-122 increased serum levels in HCC patients compared to healthy controls. MiR-122 levels were also significantly higher in HBV compared to non-HBV-related individuals, although the etiology of non-HBV patients was not specified [75].

Trung et al. focused on patients with HBV-related liver disease and found a progressive increase in circulating miR-122 expression levels from chronic HBV to liver cirrhosis up to HCC. In addition, they evaluated the diagnostic performance of a biomarker panel based on AFP levels, serum expression of miR-122 and circulating cell-free DNA mutations in the promoter of the Telomerase Reverse Transcriptase (*TERT*) gene, for distinguishing the different groups of HBV-related liver disease patients. Compared to the performance achieved by using single/double biomarkers, the triple combination panel showed the best diagnostic accuracy in discriminating HCC from chronic HBV (AUC = 0.98), cirrhosis (AUC = 0.88) or both (chronic HBV + cirrhosis, AUC = 0.94) [76].

Regarding HCV-related HCC cases, Elfert et al. found that expression levels of miR-122 were significantly higher in serum of the HCV-related HCC group than healthy donors, and also that circulating miR-122 was decreased in the HCV-related HCC group when compared to HCV patients [45].

El-Ahwany et al. analyzed circulating miR-122 expression levels in HCV genotype 4 Egyptian patients at different stages of liver disease (HCV with early fibrosis, HCV with cirrhosis and HCV-related late stage HCC) and in healthy controls. They found not only an upregulation of miR-122 in all groups of HCV patients compared to controls (as described above) but also a downregulation of miR-122 in HCV-related HCC compared to all other study groups. MiR-122 also showed good diagnostic potential in discriminating HCC patients [64].

Taken together, these studies suggest a different behavior of miR-122 in HBV-related and HCV-related HCC patients: in the first case, a gradual increase in miR-122 expression levels was observed in relation to liver disease progression, while in the second, miRNA-122 seems to be decreased in HCV-related HCC patients compared to HCV patients, suggesting that circulating miR-122 levels significantly increase during hepatocyte damage and subsequently decrease once the liver enters carcinogenesis [45].

Conversely, Franck et al., analyzing a European cohort of HCC patients, found no differences in miR-122 expression levels in relation to the progression of liver damage (disease stage and cirrhosis) and the underlying HCC etiology, which included alcohol abuse (n = 41), viral hepatitis (n = 12), non-alcoholic steatohepatitis (n = 13), hemochromatosis (n = 6) and rare or other (n = 19) [77].

In addition to its potential role as a diagnostic biomarker in HCC, the currently available literature provided case studies regarding the possibility of using miR-122 as a prognostic and predictive biomarker as well.

Pelizzaro et al. found that miR-122 expression levels, measured before TACE treatment, were predictive of HCC progression-free survival [Hazard Ratio (HR) 2.98 (1.10–8.09), *p* = 0.03] [74].

Similarly, Franck et al. observed a trend towards a better overall survival in HCC patients with low miR-122 expression levels, while statistically significant results were obtained considering the prognostic value of miR-122 in specific HCC subgroups (patients with advanced cirrhosis, advanced tumor stage and normal AFP) [77].

On the other hand, Pinjaroen et al. analyzed circulating pre-treatment expression levels of miR-122, miR-221 and miR-224 in 100 HBV-related HCC patients treated with TACE to clarify their possible role in predicting patients’ outcome. The authors identified high miR-221 expression levels and advanced HCC staging as independent predictive factors of TACE refractoriness and short overall survival, while no significant findings emerged for miR-122 and miR-224 [78].

In another study, Wu et al. found an association between higher miR-122 expression levels and the overall survival of HCC patients [HR 0.43 (0.27–0.7), log rank *p* = 0.0004], with an opposite trend to that described by Franck et al. Interestingly, no significant differences in miR-122 expression levels were found in HCC compared to heathy controls [79].

An overview of the main studies that analyzed circulating levels of miR-122 in liver diseases is provided in Table 3.

## 8. Conclusions and Future Directions

MiR-122 is deeply involved in the development of liver disease and the literature data are quite consistent on the downregulation of its tissue expression levels as well as the upregulation of serum levels following liver damage.

High circulating levels of miR-122 appear to correlate with early liver injury in both metabolic disorders and viral infection. Moreover, miR-122 shows an excellent diagnostic accuracy in discriminating patients from healthy controls, making this miRNA a highly sensitive but not very specific early diagnostic biomarker of liver disease. On the other hand, due to the dynamic nature of circulating miR-122 expression levels, controversial data are currently available on the potential use of this miRNA as a prognostic marker of liver disease progression and survival.

MiR-122 appears to be progressively upregulated in sera of patients at different stages of liver disease, as observed by comparing HBV and HBV-related HCC patients, while an opposite trend was reported by comparing HCV and HCV-related HCC patients.

Therefore, although circulating miR-122 levels are generally increased in HCC patients compared to healthy controls, the different etiology of HCC seems to have a strong impact on modulating the release of this miRNA during liver disease progression, suggesting an etiology-dependent dysregulation of miR-122 circulating levels.

In this context, the use of miRNA panels, comprising miR-122, or the combination of circulating miR-122 with conventional diagnostic markers/strategies could not only improve diagnostic performance but also increase specificity for different liver diseases, including HCC.

Overall, the importance of circulating miR-122 should be further explored by performing more comprehensive studies involving larger numbers of patients at different stages of liver disease characterized by different etiologies, possibly analyzing both tissue and plasma/serum samples. Despite its high potential, especially as a diagnostic biomarker, miR-122 should be employed in combination with other miRNAs and protein biomarkers to achieve more specific and greater diagnostic accuracy for different types of liver diseases.

## Figures and Tables

**Table 1:** Main methods available for liver disease diagnosis and related advantages and disadvantages.

Diagnostic Method	Liver Disease	Pro	Cons
Serum transaminases	ALD	Non-invasive. High sensitivity (particularly for the diagnosis of chronic alcohol abuse)	Low specificity (no information on the type of liver injury)
	NAFLD		
	HBV/HCV		
	HCC		
Liver biopsy	NAFLD	Clear diagnosis of liver injury	Invasive method. Possible relevant complications
	HBV/HCV		
	HCC		
FIB-4 index and NFS	NAFLD	Non-invasive and time-saving	Low specificity
MRI-PDFF		Non-invasive. Like biopsy in term of results	High cost and time consuming
Viral antigen	HBV/HCV	Non-invasive. High sensitivity and specificity	Useful during viral incubation only
Viral load			Useful during chronic viral infection only
AFP	HCC	Non-invasive	Limited sensitivity and specificity

**Table 2:** Overview of the molecular characteristics and main biological functions of miR-122.

hsa-miR-122	
**Full name**	microRNA 122
**Symbol**	MIR122
**Alias**	miR-122a; miR-122-5p
**Location**	Chromosome 18q21.31
**Length**	
**pri-miR-122**	4.5 kb
**pre-miR-122**	66 nt
**mature miR-122**	22 nt
**Other information**	Conserved sequence across species, no known paralogs of the mature miR-122
**Main biological functions**	Hepatocyte differentiation; lipogenesis; ion/amino acid transport; inflammation; viral replication, cell proliferation, migration and epithelial–mesenchymal transition

**Table 3:** Table showing the characteristics of the main studies described in this review about circulating levels of miR-122 in different liver diseases and its role as putative diagnostic, prognostic and predictive biomarker.

	Study Type	Cases	Controls	MiR-122 Expression	MiR-122 Performance Parameters/Association with Clinico-Pathological Features	Suggested Combination with Other Biomarkers	References
**NAFLD diagnosis**	Systematic review and Meta-analysis	NAFLD patients	Healthy controls	Upregulated	AUC 0.86 (95% CI 0.82–0.88), SE 0.66, SP 0.88		[49]
	Systematic review	NAFLD/insulin-resistant obese children	Normal-weight non-NAFLD children/obese children with insulin sensitivity	Upregulated		miR-122, miR-34a	[50]
	Article	Obese adolescents w/NAFLD	Obese adolescents without NAFLD	Upregulated	Positive association with histological features of NAFLD	miR-122, miR-1343-5p, miR-193a-5p, miR-193b-5p, and miR-7845-5p	[51]
	Article	Obese women with high fasting blood glucose with/without signs of fatty liver infiltration	Healthy controls	Upregulated		miR-122, miR-34a, miR-375, miR-16, miR-21, IL-6, Leptin, FABP-1	[52]
	Article	NAFLD mouse model/NAFLD patients	Healthy controls	Upregulated	AUC 0.79 (95% CI, 0.70–0.88)		[53]
**NAFLD diagnosis/progression**	Article	NAFL patients	Healthy controls	Upregulated	AUC 0.67 (95% CI, 0.56–0.78), SE 0.68, SP 0.70, *p* < 0.005	miR-122, miR-128, miR-200, miR-298, miR-342	[44]
		NASH patients			AUC 1.00 (95% CI: 1.00–1.00), SE 1.00, SP 1.00, *p* < 0.001		
		NASH patients	NAFL patients				
**NAFLD progression**	Article	NAFLD patients		Upregulated	Positive association with features of NAFLD severity	miR-122, miR-34a, miR-192, miR-200a	[54]
	Article	Apoe^−/−^ HFD mice	Standard diet-fed WT mice	Upregulated		miR-122, miR-27b-3p	[55]
	Article	ASGR1-EVs from NASH patients	Healthy controls	Upregulated	AUC 0.83, *p* = 0.004 (all NAFLD patients vs. controls)	miR-122, miR-192, miR-128-3p	[56]
		ASGR1-EVs from NAFL, global EVs or total cell-free RNA from NAFL and NASH patients		No difference			
**NAFLD prognosis (survival rate)**	Article	NAFLD patients-low miR-122 expression)	NAFLD patients, high miR-122 expression		Worst cumulative survival rate [<1.00 fold change; HR 4.35 (95% CI, 0.06–0.83), *p* = 0.025]	miR-122, FIB-4 index	[58]
**ALD diagnosis**	Meta-analysis	ALD patients	Healthy controls	Upregulated			[59]
**HCV diagnosis**	Meta-analysis	HCV-associated chronic hepatitis patients	Healthy controls	Upregulated	AUC 0.95 (95% CI, 0.93–0.97), SE 0.94 (95%CI, 0.89–0.97), SP 0.85 (95% CI, 0.78–0.90)		[60]
**HBV diagnosis**		HBV-associated chronic hepatitis patients			AUC 0.88 (95% CI, 0.85–0.91), SE 0.87 (95%CI, 0.75–0.94), SP 0.81 (95% CI, 0.75–0.87)		
**HCV diagnosis**	Article	HCV patients	Healthy controls	Upregulated	AUC 0.97 (95% CI 0.93–1.02), *p* < 0.001	miR-122, miR-483, miR-335	[45]
**HCC diagnosis**		HCV-related HCC patients			AUC 0.95 (95% CI 0.90–1.02), *p* < 0.0001		
		HCV-related HCC patients	HCV patients	Downregulated	AUC 0.85 (95% CI 0.74–0.97), *p* < 0.0001		
**HCV diagnosis/progression**	Article	HCV patients (genotype-3) with mild, moderate, severe cirrhosis	Healthy controls	Upregulated (no differences among the three case groups)	mean AUC 0.82, *p* < 0.0001		[47]
**HCV diagnosis/progression**	Article	HCV patients (early fibrosis)	Healthy controls	Upregulated (no difference between the two case groups)		miR-122, miR-125a, miR-139, miR-145, miR-199a	[64]
		HCV patients with cirrhosis					
**HCC diagnosis**		HCV-related HCC patients		Downregulated	AUC 0.90 (95% CI 0.84–0.95), SE 0.85, SP 0.83		
			HCV patients (early fibrosis)				
			HCV patients (cirrhosis)				
**HCV diagnosis/treatment response prediction**	Article	Chronic HCV (genotype-4) patients	Healthy controls	Upregulated	AUC 0.94 (95%CI 0.92–0.97), SE 0.83, SP 1.00, *p* < 0.001	miR-122, miR-155, miR-196, miR-29	[65]
		Chronic HCV (genotype-4) patients, therapy responders	Chronic HCV genotype-4 patients, therapy non-responders	Downregulated	AUC 0.97 (95%CI 0.93–0.99), SE 0.86, SP 1.00, *p* < 0.0001	miR-122, miR-155, miR-196, miR-29	
**HBV diagnosis**	Article	Chronic HBV patients	HBeAg-neg HBV patients	Upregulated			[69]
	Article	Chronic HBV patients (active and inactive)	Healthy controls	Upregulated	AUC 0.89, SE 1.00, SP 0.62, *p* < 0.0001	miR-122, miR-583	[70]
		Inactive HBV carriers	Active HBV patients	No difference			
**HCC diagnosis**	Meta-analysis	HCC patients	Healthy controls		AUC 0.91 (95% CI 0.88–0.93), SE 0.85, SP 0.83, *p* < 0.01		[72]
			HCV/HBV patients		AUC 0.87 (95% CI 0.84–0.90), SE 0.79, SP 0.83, *p* < 0.01		
			Liver cirrhosis or dysplastic nodule formation		AUC 0.74 (95% CI 0.69–0.77), SE 0.65, SP 0.75, *p* < 0.01		
**HCC diagnosis**	Article	early-stage HCC patients	Controls (HBV patients, liver cirrhosis, liver metastases, hepatic hemangioma and healthy individuals)	Upregulated (progressive increase from healthy individuals to HBV up to HCC patients)	AUC 0.76 (95% CI, 0.69–0.83), SE 0.83, SP 0.64, *p* < 0.001	miR-122, miR-16, AFP	[73]
	Article	HCC patients	Healthy controls and cirrhotic patients	Upregulated (progressive increase from healthy controls to cirrhosis up to HCC)			[74]
		HCV/HBV-related HCC patients	Other HCC etiology patients	Upregulated			
**HCC prognosis (survival rate)**		HCC patients (low miR-122 expression)	HCC patients (high miR-122 expression)		Better progression-free survival [HR 2.98 (1.10–8.09), logrank *p* = 0.03]		
**HCC diagnosis**	Article	HBV-related HCC patients	Chronic HBV and HBV-related cirrhosis patients	Upregulated (progressive increase from chronic HBV to cirrhosis up to HCC)		miR-122, AFP, cell-free DNA *TERT* gene mutations	[76]
**HCC prognosis (progression/survival rate)**	Article	HCC patients		No difference in relation to tumor progression (disease stage and cirrhosis) and HCC etiology			[77]
		HCC patients (low miR-122 expression)	HCC patients (high miR-122 expression)		Trend towards a better overall survival		
**HCC prognosis/treatment response prediction**	Article	HBV-related HCC patients			No association with treatment refractoriness and overall survival		[78]
**HCC diagnosis/prognosis**	Article	HCC patients	Healthy controls	No difference			[79]
		HCC patients (high miR-122 expression)	HCC patients (low miR-122 expression)		Better overall survival [HR 0.43 (0.27–0.7), logrank *p* = 0.0004]		

Abbreviations: SE, sensitivity; SP, specificity; AUC, Area Under The Curve; 95%CI, confidence interval; HR, hazard ratio. Each color indicates a different liver disease (NAFLD, ALD, HCV, HBV, HCC).

## Data Availability

No new data were created or analyzed in this study. Data sharing is not applicable to this article.

## References

[1] O’Brien J., Hayder H., Zayed Y., Peng C. (2018). Overview of MicroRNA Biogenesis, Mechanisms of Actions, and Circulation. Front. Endocrinol..

[2] Zelli V., Compagnoni C., Capelli R., Corrente A., Di Vito Nolfi M., Zazzeroni F., Alesse E., Tessitore A. (2022). Role of Exosomal microRNAs in Cancer Therapy and Drug Resistance Mechanisms: Focus on Hepatocellular Carcinoma. Front. Oncol..

[3] Hochreuter M.Y., Dall M., Treebak J.T., Barrès R. (2022). MicroRNAs in Non-Alcoholic Fatty Liver Disease: Progress and Perspectives. Mol. Metab..

[4] Musaddaq G., Shahzad N., Ashraf M.A., Arshad M.I. (2019). Circulating Liver-Specific microRNAs as Noninvasive Diagnostic Biomarkers of Hepatic Diseases in Human. Biomark. Biochem. Indic. Expo. Response Susceptibility Chem..

[5] Faramin Lashkarian M., Hashemipour N., Niaraki N., Soghala S., Moradi A., Sarhangi S., Hatami M., Aghaei-Zarch F., Khosravifar M., Mohammadzadeh A. (2023). MicroRNA-122 in Human Cancers: From Mechanistic to Clinical Perspectives. Cancer Cell Int..

[6] Chang J., Nicolas E., Marks D., Sander C., Lerro A., Buendia M.A., Xu C., Mason W.S., Moloshok T., Bort R. (2004). miR-122, a Mammalian Liver-Specific microRNA, Is Processed from Hcr mRNA and May Downregulate the High Affinity Cationic Amino Acid Transporter CAT-1. RNA Biol..

[7] Schult P., Roth H., Adams R.L., Mas C., Imbert L., Orlik C., Ruggieri A., Pyle A.M., Lohmann V. (2018). microRNA-122 Amplifies Hepatitis C Virus Translation by Shaping the Structure of the Internal Ribosomal Entry Site. Nat. Commun..

[8] Long J.-K., Dai W., Zheng Y.-W., Zhao S.-P. (2019). miR-122 Promotes Hepatic Lipogenesis via Inhibiting the LKB1/AMPK Pathway by Targeting Sirt1 in Non-Alcoholic Fatty Liver Disease. Mol. Med..

[9] Singal A.G., Kanwal F., Llovet J.M. (2023). Global Trends in Hepatocellular Carcinoma Epidemiology: Implications for Screening, Prevention and Therapy. Nat. Rev. Clin. Oncol..

[10] Rumgay H., Arnold M., Ferlay J., Lesi O., Cabasag C.J., Vignat J., Laversanne M., McGlynn K.A., Soerjomataram I. (2022). Global Burden of Primary Liver Cancer in 2020 and Predictions to 2040. J. Hepatol..

[11] Calderon-Martinez E., Landazuri-Navas S., Vilchez E., Cantu-Hernandez R., Mosquera-Moscoso J., Encalada S., Al Lami Z., Zevallos-Delgado C., Cinicola J. (2023). Prognostic Scores and Survival Rates by Etiology of Hepatocellular Carcinoma: A Review. J. Clin. Med. Res..

[12] Younossi Z.M., Wong G., Anstee Q.M., Henry L. (2023). The Global Burden of Liver Disease. Clin. Gastroenterol. Hepatol. Off. Clin. Pract. J. Am. Gastroenterol. Assoc..

[13] Toh M.R., Wong E.Y.T., Wong S.H., Ng A.W.T., Loo L.-H., Chow P.K.-H., Ngeow J. (2023). Global Epidemiology and Genetics of Hepatocellular Carcinoma. Gastroenterology.

[14] Huang D.Q., Mathurin P., Cortez-Pinto H., Loomba R. (2023). Global Epidemiology of Alcohol-Associated Cirrhosis and HCC: Trends, Projections and Risk Factors. Nat. Rev. Gastroenterol. Hepatol..

[15] Vargas M., Cardoso Toniasso S.d.C., Riedel P.G., Baldin C.P., Dos Reis F.L., Pereira R.M., Brum M.C.B., Joveleviths D., Alvares-da-Silva M.R. (2024). Metabolic Disease and the Liver: A Review. World J. Hepatol..

[16] Ginès P., Krag A., Abraldes J.G., Solà E., Fabrellas N., Kamath P.S. (2021). Liver Cirrhosis. Lancet.

[17] Kong L.-Z., Chandimali N., Han Y.-H., Lee D.-H., Kim J.-S., Kim S.-U., Kim T.-D., Jeong D.K., Sun H.-N., Lee D.S. (2019). Pathogenesis, Early Diagnosis, and Therapeutic Management of Alcoholic Liver Disease. Int. J. Mol. Sci..

[18] Pouwels S., Sakran N., Graham Y., Leal A., Pintar T., Yang W., Kassir R., Singhal R., Mahawar K., Ramnarain D. (2022). Non-Alcoholic Fatty Liver Disease (NAFLD): A Review of Pathophysiology, Clinical Management and Effects of Weight Loss. BMC Endocr. Disord..

[19] EASL-EASD-EASO (2016). Clinical Practice Guidelines for the Management of Non-Alcoholic Fatty Liver Disease. J. Hepatol..

[20] Petzold G. (2022). Role of Ultrasound Methods for the Assessment of NAFLD. J. Clin. Med..

[21] Pezzuto F., Buonaguro L., Buonaguro F.M., Tornesello M.L. (2018). The Role of Circulating Free DNA and MicroRNA in Non-Invasive Diagnosis of HBV- and HCV-Related Hepatocellular Carcinoma. Int. J. Mol. Sci..

[22] Singh S., Hoque S., Zekry A., Sowmya A. (2023). Radiological Diagnosis of Chronic Liver Disease and Hepatocellular Carcinoma: A Review. J. Med. Syst..

[23] Di Mauro S., Scamporrino A., Filippello A., Di Pino A., Scicali R., Malaguarnera R., Purrello F., Piro S. (2021). Clinical and Molecular Biomarkers for Diagnosis and Staging of NAFLD. Int. J. Mol. Sci..

[24] Easterbrook P.J., Roberts T., Sands A., Peeling R. (2017). Diagnosis of Viral Hepatitis. Curr. Opin. HIV AIDS.

[25] Boonkaew B., Satthawiwat N., Pinjaroen N., Chuaypen N., Tangkijvanich P. (2023). Circulating Extracellular Vesicle-Derived microRNAs as Novel Diagnostic and Prognostic Biomarkers for Non-Viral-Related Hepatocellular Carcinoma. Int. J. Mol. Sci..

[26] Atic A.I., Thiele M., Munk A., Dalgaard L.T. (2023). Circulating miRNAs Associated with Nonalcoholic Fatty Liver Disease. Am. J. Physiol. Cell Physiol..

[27] Thakral S., Ghoshal K. (2015). miR-122 Is a Unique Molecule with Great Potential in Diagnosis, Prognosis of Liver Disease, and Therapy Both as miRNA Mimic and Antimir. Curr. Gene Ther..

[28] Chou C.-H., Shrestha S., Yang C.-D., Chang N.-W., Lin Y.-L., Liao K.-W., Huang W.-C., Sun T.-H., Tu S.-J., Lee W.-H. (2018). miRTarBase Update 2018: A Resource for Experimentally Validated microRNA-Target Interactions. Nucleic Acids Res..

[29] Bartel D.P. (2004). MicroRNAs: Genomics, Biogenesis, Mechanism, and Function. Cell.

[30] Krek A., Grün D., Poy M.N., Wolf R., Rosenberg L., Epstein E.J., MacMenamin P., da Piedade I., Gunsalus K.C., Stoffel M. (2005). Combinatorial microRNA Target Predictions. Nat. Genet..

[31] Suzuki H., Maruyama R., Yamamoto E., Kai M. (2013). Epigenetic Alteration and microRNA Dysregulation in Cancer. Front. Genet..

[32] Davis-Dusenbery B.N., Hata A. (2010). MicroRNA in Cancer: The Involvement of Aberrant MicroRNA Biogenesis Regulatory Pathways. Genes Cancer.

[33] Hsu S.-H., Wang B., Kota J., Yu J., Costinean S., Kutay H., Yu L., Bai S., La Perle K., Chivukula R.R. (2012). Essential Metabolic, Anti-Inflammatory, and Anti-Tumorigenic Functions of miR-122 in Liver. J. Clin. Investig..

[34] Davoodian N., Lotfi A.S., Soleimani M., Mowla S.J. (2014). MicroRNA-122 Overexpression Promotes Hepatic Differentiation of Human Adipose Tissue-Derived Stem Cells. J. Cell. Biochem..

[35] Krützfeldt J., Rajewsky N., Braich R., Rajeev K.G., Tuschl T., Manoharan M., Stoffel M. (2005). Silencing of microRNAs in Vivo with “Antagomirs”. Nature.

[36] Niu Y., Lin Z., Wan A., Sun L., Yan S., Liang H., Zhan S., Chen D., Bu X., Liu P. (2021). Loss-of-Function Genetic Screening Identifies Aldolase A as an Essential Driver for Liver Cancer Cell Growth Under Hypoxia. Hepatology.

[37] Li X.-N., Yang H., Yang T. (2020). miR-122 Inhibits Hepatocarcinoma Cell Progression by Targeting LMNB2. Oncol. Res..

[38] Bai S., Nasser M.W., Wang B., Hsu S.-H., Datta J., Kutay H., Yadav A., Nuovo G., Kumar P., Ghoshal K. (2009). MicroRNA-122 Inhibits Tumorigenic Properties of Hepatocellular Carcinoma Cells and Sensitizes These Cells to Sorafenib. J. Biol. Chem..

[39] Cheng D., Deng J., Zhang B., He X., Meng Z., Li G., Ye H., Zheng S., Wei L., Deng X. (2018). LncRNA HOTAIR Epigenetically Suppresses miR-122 Expression in Hepatocellular Carcinoma via DNA Methylation. eBioMedicine.

[40] Wen D.-Y., Huang J.-C., Wang J.-Y., Pan W.-Y., Zeng J.-H., Pang Y.-Y., Yang H. (2018). Potential Clinical Value and Putative Biological Function of miR-122-5p in Hepatocellular Carcinoma: A Comprehensive Study Using Microarray and RNA Sequencing Data. Oncol. Lett..

[41] Fu X., Calin G.A. (2018). miR-122 and Hepatocellular Carcinoma: From Molecular Biology to Therapeutics. eBioMedicine.

[42] Qiao D.-D., Yang J., Lei X.-F., Mi G.-L., Li S.-L., Li K., Xu C.-Q., Yang H.-L. (2017). Expression of microRNA-122 and microRNA-22 in HBV-Related Liver Cancer and the Correlation with Clinical Features. Eur. Rev. Med. Pharmacol. Sci..

[43] Esau C., Davis S., Murray S.F., Yu X.X., Pandey S.K., Pear M., Watts L., Booten S.L., Graham M., McKay R. (2006). miR-122 Regulation of Lipid Metabolism Revealed by in Vivo Antisense Targeting. Cell Metab..

[44] Samy A.M., Kandeil M.A., Sabry D., Abdel-Ghany A.A., Mahmoud M.O. (2024). Exosomal miR-122, miR-128, miR-200, miR-298, and miR-342 as Novel Diagnostic Biomarkers in NAFL/NASH: Impact of LPS/TLR-4/FoxO3 Pathway. Arch. Pharm..

[45] Elfert A.Y., Salem A., Abdelhamid A.M., Salama A., Sourour D.A., Shaker O., Keshk M. (2022). Implication of miR-122, miR-483, and miR-335 Expression Levels as Potential Signatures in HCV-Related Hepatocellular Carcinoma (HCC) in Egyptian Patients. Front. Mol. Biosci..

[46] Gaber D.A., Shaker O., Younis A.T., El-Kassas M. (2022). LncRNA HULC and miR-122 Expression Pattern in HCC-Related HCV Egyptian Patients. Genes.

[47] Ullah A., Yu X., Odenthal M., Meemboor S., Ahmad B., Rehman I.U., Ahmad J., Ali Q., Nadeem T. (2022). Circulating microRNA-122 in HCV Cirrhotic Patients with High Frequency of Genotype 3. PLoS ONE.

[48] Powell E.E., Wong V.W.-S., Rinella M. (2021). Non-Alcoholic Fatty Liver Disease. Lancet.

[49] Cai C., Lin Y., Yu C. (2019). Circulating miRNAs as Novel Diagnostic Biomarkers in Nonalcoholic Fatty Liver Disease: A Systematic Review and Meta-Analysis. Can. J. Gastroenterol. Hepatol..

[50] Oses M., Margareto Sanchez J., Portillo M.P., Aguilera C.M., Labayen I. (2019). Circulating miRNAs as Biomarkers of Obesity and Obesity-Associated Comorbidities in Children and Adolescents: A Systematic Review. Nutrients.

[51] Li Y.-J., Baumert B.O., Stratakis N., Goodrich J.A., Wu H.-T., He J.-X., Zhao Y.-Q., Aung M.T., Wang H.-X., Eckel S.P. (2024). Circulating microRNA Expression and Nonalcoholic Fatty Liver Disease in Adolescents with Severe Obesity. World J. Gastroenterol..

[52] Pillai S.S., Lakhani H.V., Zehra M., Wang J., Dilip A., Puri N., O’Hanlon K., Sodhi K. (2020). Predicting Nonalcoholic Fatty Liver Disease through a Panel of Plasma Biomarkers and MicroRNAs in Female West Virginia Population. Int. J. Mol. Sci..

[53] Jiang H., Qian Y., Shen Z., Liu Y., He Y., Gao R., Shen M., Chen S., Fu Q., Yang T. (2021). Circulating microRNA-135a-3p in Serum Extracellular Vesicles as a Potential Biological Marker of Non-alcoholic Fatty Liver Disease. Mol. Med. Rep..

[54] Ezaz G., Trivedi H.D., Connelly M.A., Filozof C., Howard K., L Parrish M., Kim M., Herman M.A., Nasser I., Afdhal N.H. (2020). Differential Associations of Circulating MicroRNAs with Pathogenic Factors in NAFLD. Hepatol. Commun..

[55] López-Pastor A.R., Infante-Menéndez J., González-Illanes T., González-López P., González-Rodríguez Á., García-Monzón C., Vega de Céniga M., Esparza L., Gómez-Hernández A., Escribano Ó. (2021). Concerted Regulation of Non-Alcoholic Fatty Liver Disease Progression by microRNAs in Apolipoprotein E-Deficient Mice. Dis. Model. Mech..

[56] Newman L.A., Useckaite Z., Johnson J., Sorich M.J., Hopkins A.M., Rowland A. (2022). Selective Isolation of Liver-Derived Extracellular Vesicles Redefines Performance of miRNA Biomarkers for Non-Alcoholic Fatty Liver Disease. Biomedicines.

[57] Akuta N., Kawamura Y., Suzuki F., Saitoh S., Arase Y., Muraishi N., Fujiyama S., Sezaki H., Hosaka T., Kobayashi M. (2022). Dynamics of Circulating miR-122 Predict Liver Cancer and Mortality in Japanese Patients with Histopathologically Confirmed NAFLD and Severe Fibrosis Stage. Oncology.

[58] Akuta N., Kawamura Y., Arase Y., Saitoh S., Fujiyama S., Sezaki H., Hosaka T., Kobayashi M., Kobayashi M., Suzuki Y. (2020). Circulating MicroRNA-122 and Fibrosis Stage Predict Mortality of Japanese Patients with Histopathologically Confirmed NAFLD. Hepatol. Commun..

[59] Hu T., Liu C.H., Zheng Y., Ji J., Zheng Y., He S.-K., Wu D., Jiang W., Zeng Q., Zhang N. (2024). miRNAs in Patients with Alcoholic Liver Disease: A Systematic Review and Meta-Analysis. Expert Rev. Gastroenterol. Hepatol..

[60] Zhou X., Fang S., Wang M., Xiong A., Zheng C., Wang J., Yin C. (2019). Diagnostic Value of Circulating miRNA-122 for Hepatitis B Virus and/or Hepatitis C Virus-Associated Chronic Viral Hepatitis. Biosci. Rep..

[61] Moayedi J., Hashempour T., Musavi Z., Arefian E., Naderi M., Heidari M.R., Dehghani B., Hasanshahi Z., Merat S. (2021). Evaluation of miR-122 Serum Level and IFN-Λ3 Genotypes in Patients with Chronic HCV and HCV-Infected Liver Transplant Candidate. MicroRNA.

[62] El-Hefny M., Fouad S., Hussein T., Abdel-Hameed R., Effat H., Mohamed H., Abdel Wahab A.H. (2019). Circulating microRNAs as Predictive Biomarkers for Liver Disease Progression of Chronic Hepatitis C (Genotype-4) Egyptian Patients. J. Med. Virol..

[63] Al-Rawaf H.A., Gabr S.A., Iqbal A., Alghadir A.H. (2024). Circulating microRNAs as Potential Biomarkers of Physical Activity in Geriatric Patients with HCV. BMC Mol. Cell Biol..

[64] El-Ahwany E.G.E., Mourad L., Zoheiry M.M.K., Abu-Taleb H., Hassan M., Atta R., Hassanien M., Zada S. (2019). MicroRNA-122a as a Non-Invasive Biomarker for HCV Genotype 4-Related Hepatocellular Carcinoma in Egyptian Patients. Arch. Med. Sci. AMS.

[65] Hassuna N.A., Gamil A.N., Mahmoud M.S., Mohamed W.K., Khairy R. (2022). Circulating microRNAs as Predictors of Response to Sofosbuvir + Daclatasvir + Ribavirin in in HCV Genotype-4 Egyptian Patients. BMC Gastroenterol..

[66] Ezzat W.M., Amr K.S., Tawfeek S., Elbatae H., Bayomi E.A., Heiba A., Elhosary Y. (2024). Serum MicroRNA Profiles in Chronic Hepatitis C Egyptian Patients before and after Combined Sofosbuvir and Daclatasvir Treatment. BMC Infect. Dis..

[67] Franco S., Llibre J.M., Jou T., Tural C., Martínez M.A. (2023). Normalization of Circulating Plasma Levels of miRNAs in HIV-1/HCV Co-Infected Patients Following Direct-Acting Antiviral-Induced Sustained Virologic Response. Heliyon.

[68] Muthukumar T., Akat K.M., Yang H., Schwartz J.E., Li C., Bang H., Ben-Dov I.Z., Lee J.R., Ikle D., Demetris A.J. (2022). Serum MicroRNA Transcriptomics and Acute Rejection or Recurrent Hepatitis C Virus in Human Liver Allograft Recipients: A Pilot Study. Transplantation.

[69] Cavallone D., Ornos E.D.B., Ricco G., Oliveri F., Coco B., Colombatto P., De Rosa L., Dalmacio L.M.M., Bonino F., Brunetto M.R. (2023). The Circulating miRNA Profile of Chronic Hepatitis D and B Patients Is Comparable but Differs from That of Individuals with HBeAg-Negative HBV Infection. Viruses.

[70] Mokhtari F., Kaboosi H., Mohebbi S.R., Asadzadeh Aghdaei H., Zali M.R. (2023). Circulating Plasma miR-122 and miR-583 Levels Are Involved in Chronic Hepatitis B Virus Pathogenesis and Serve As Novel Diagnostic Biomarkers. Genet. Test. Mol. Biomark..

[71] Liu W., He X., Huang F. (2021). Analysis of Serum MicroRNA-122 Expression at Different Stages of Chronic Hepatitis B Virus Infection. BioMed Res. Int..

[72] Zhao X.-F., Li N., Lin D.-D., Sun L.-B. (2020). Circulating MicroRNA-122 for the Diagnosis of Hepatocellular Carcinoma: A Meta-Analysis. BioMed Res. Int..

[73] Fang Y., Yan D., Wang L., Zhang J., He Q. (2022). Circulating microRNAs (miR-16, miR-22, miR-122) Expression and Early Diagnosis of Hepatocellular Carcinoma. J. Clin. Lab. Anal..

[74] Pelizzaro F., Cardin R., Sartori A., Imondi A., Penzo B., Aliberti C., Ponzoni A., Vitale A., Cillo U., Farinati F. (2021). Circulating MicroRNA-21 and MicroRNA-122 as Prognostic Biomarkers in Hepatocellular Carcinoma Patients Treated with Transarterial Chemoembolization. Biomedicines.

[75] Mjelle R., Dima S.O., Bacalbasa N., Chawla K., Sorop A., Cucu D., Herlea V., Sætrom P., Popescu I. (2019). Comprehensive Transcriptomic Analyses of Tissue, Serum, and Serum Exosomes from Hepatocellular Carcinoma Patients. BMC Cancer.

[76] Trung N.T., Hoan N.X., Trung P.Q., Binh M.T., Van Tong H., Toan N.L., Bang M.H., Song L.H. (2020). Clinical Significance of Combined Circulating TERT Promoter Mutations and miR-122 Expression for Screening HBV-Related Hepatocellular Carcinoma. Sci. Rep..

[77] Franck M., Schütte K., Malfertheiner P., Link A. (2020). Prognostic Value of Serum microRNA-122 in Hepatocellular Carcinoma Is Dependent on Coexisting Clinical and Laboratory Factors. World J. Gastroenterol..

[78] Pinjaroen N., Chailapakul P., Sriphoosanaphan S., Chuaypen N., Tangkijvanich P. (2023). Predictive Role of Pretreatment Circulating miR-221 in Patients with Hepatocellular Carcinoma Undergoing Transarterial Chemoembolization. Diagnostics.

[79] Wu J., Wu Y., Luo Y., Li X., Lin N., Yang X., Lin Y., Chen M. (2020). Circulating miRNA-199a and miRNA-122 Levels as Potential Diagnostic and Prognostic Biomarkers for Hepatocellular Carcinoma. Ann. Clin. Lab. Sci..

